# Application of *Lactobacillus helveticus* KLDS 1.1105 Postbiotics for Resisting Pathogenic Bacteria Infection in the Intestine

**DOI:** 10.3390/foods14152659

**Published:** 2025-07-29

**Authors:** Peng Du, Jiaying Liu, Chengwen Hu, Jianing Zhang, Miao Li, Yu Xin, Libo Liu, Aili Li, Chun Li

**Affiliations:** 1 Key Laboratory of Dairy Science, Ministry of Education, Northeast Agricultural University (NEAU), Harbin 150030, China; dupeng@neau.edu.cn (P.D.); l17753622906@163.com (J.L.); hu10492758@163.com (C.H.); liboliu@126.com (L.L.); 2 Heilongjiang Green Food Science Research Institute, Northeast Agricultural University (NEAU), Harbin 150028, China; 3 College of Food Science, Northeast Agricultural University (NEAU), Harbin 150030, China

**Keywords:** *Lactobacillus helveticus* postbiotics (*LHPs*), SHIME, probiotic, intestinal flora, SCFAs

## Abstract

Postbiotics, defined as metabolites produced by probiotics, encompass both bacterial cells and their metabolic byproducts, and offer significant health benefits to the host. However, there are relatively few reports on their effects on intestinal microbiota. In this study, we investigated the components, total antioxidant capacity of *Lactobacillus helveticus* postbiotics (*LHPs*) and their impact on intestinal flora using the Simulator for Human Intestinal Microecology Simulation (SHIME). The results indicate that the primary components of postbiotics include polysaccharides, proteins, and organic acids. Furthermore, *LHPs* have a strong ability to inhibit the growth of harmful bacteria while promoting the growth of probiotics. Additionally, *LHPs* significantly increased the total antioxidant capacity in the intestine and regulated the balance of intestinal microbiota. Notably, there was also a significant increase in the content of short-chain fatty acids (SCFAs) in the intestine. Overall, *LHPs* have the potential to aid in the prevention and treatment of diseases by enhancing gut microbiology.

## 1. Introduction

In recent years, research has increasingly focused on the relationship between beneficial microorganisms and the human gut, emphasizing three primary methods for regulating intestinal flora: probiotics, prebiotics, and synbiotics [1,2]. These concepts have gained widespread acceptance in the food and healthcare sectors. The International Scientific Association for Probiotics and Prebiotics (ISAPP) defined postbiotics as microbial agents or metabolites derived from inactivated bacteria that promote human health [3,4].

Postbiotics, produced through substrate fermentation, offer several advantages, such as enhanced safety and stability, compared to probiotics, making them easier to preserve and transport [5,6]. Postbiotics are non-living microorganisms and their components produced through the metabolism of probiotics, thereby avoiding risks associated with live probiotics, such as the potential for infection in immunocompromised populations or the transfer of antibiotic resistance genes [7]. A systematic review and meta-analysis of the addition of postbiotics to infant formulas indicates that postbiotics are associated with good safety [8]. The stability of postbiotics simplifies storage and transportation processes; reduces dependence on cold chain logistics, thereby lowering costs; and makes the products easier to promote and apply [9]. Certain postbiotics, including those from *Bifidobacterium breve* C50 and *Streptococcus salivarius* subsp. *thermophilus* 065, have been shown to positively influence gut flora composition and reduce the risk of diarrhea [10]. Additionally, fermentation of *Lactiplantibacillus plantarum* can yield postbiotics with strong antioxidant properties. A combination of specific prebiotics may also help mimic the gut microbial composition of breastfed infants [11]. *L. helveticus* is a significant fermenter used in cheese production due to its adaptability to industrial conditions and its potential for postbiotics production [12]. Fermentation with various media can increase the concentrations of amino acids and organic acids, further contributing to its value [13,14]. Research on biomass and gut flora changes can be complex and costly, often relying on in vitro simulations, particularly digestive models. Static models are limited to studying simple meals under specific conditions and may lack accuracy in representing gut dynamics [15]. In contrast, dynamic models like the SHIME provide a more realistic simulation of the human digestive process and have proven effective for studying microbial changes [16,17,18].

In this study, we aimed to analyze the main components of *LHPs* and investigate their effects on probiotic bacteria, including determining the minimum inhibitory concentration (MIC) of these postbiotics. We assessed the inhibitory properties of *LHPs* produced by different processing methods against pathogenic bacteria. Additionally, we measured the growth of various probiotics at different concentrations of *LHPs* and explored their impact on the growth characteristics of these probiotics. Furthermore, we measured the antioxidant effects of *LHPs* on human body systems using SHIME modeling. Lastly, we investigated the influence of *LHPs* on the abundance, diversity, and production of SCFAs within the human intestinal flora.

## 2. Materials and Methods

### 2.1. Materials and Chemicals

Arabinogalactan, pectin, starch, peptone, cysteine, mucin, yeast extract, and glucose were purchased from macklin (Shanghai, China). Diphenyl l-1-picrylhydrazyl (DPPH) was purchased from Sigma-Aldrich (Shanghai, China). Phenol and BCA Protein Concentration Measurement Kits were purchased from BiyunTian Biotechnology (Shanghai, China). Bile salts, lipase, α-amylase (1500 U/mg), trypsin (3000 U/mg), pepsin (3000 U/mg), papain (5000 U/mg) and catalase (1500 U/mg) were purchased from Biotopped (Beijing, China). Acid, acetic acid, propionic acid, butyric acid, isobutyric acid, and valeric acid (chromatographic grade) were purchased from Kemiou (Tianjin, China). Skimmed milk powder was purchased from Fonterra Co. (Auckland, New Zealand).

### 2.2. Bacterial Strains

*Lactobacillus helveticus* KLDS 1.1105, *Staphylococcus aureus* ATCC13565, *Escherichia coli* IQCC10126, *Lactiplantibacillus plantarum* DNZ-4, *Lacticaseibacillus casei* 0.8072, *Lacticaseibacillus rhamnosus* L08 and *Lactococcus lactis* subsp. *lactis* L1 were kindly provided by the Key Laboratory of Dairy Science, Ministry of Education, Northeast Agricultural University, China.

### 2.3. Preparation of Lactobacillus helveticus KLDS 1.1105 Postbiotic

Inoculate *L. helveticus* KLDS 1.1105 into skimmed milk for three successive generations after activation and cultivation. Monitor the strain closely for future applications. Next, prepare *LHPs* using various methods, including spray drying and freeze drying [19,20]. Spray drying of *L. helveticus* was performed under the following conditions: inlet air temperature of 150 °C to 160 °C, outlet air temperature between 45 °C and 50 °C, inlet flow rate of 7.5 mL/min, and drying air flow rate of 32.5 m^3^/h. Freeze drying of *L. helveticus* was carried out with the following parameters: freezing temperature at −40 °C, pump pressure of 100 mTorr, and shelf temperature of −60 °C.

### 2.4. Inactivation Testing of LHPs

Take 1 g of *LHPs* and dissolve it in 10 mL of sterile water within a test tube, shaking thoroughly to ensure complete dissolution. Conduct the procedure in a laminar flow hood, then aseptically transfer the *LHP* solution onto an MRS agar plate and perform streaking for isolation. Incubate the plate overnight at 37 °C in a microbial incubator.

### 2.5. Components Analysis of LPHs

The polysaccharide content was determined using the phenol–sulfuric acid method [21]. A glucose solution of a certain concentration was prepared, 1 mL of 5% phenol and 5 mL of concentrated sulfuric acid were added sequentially, followed by a 30-min incubation at room temperature. The detection wavelength was 490 nm.

Using the BCA protein concentration assay, the protein concentration standard was prepared according to the instructions and mixed with the working solution. A volume of 20 μL of the sample was added to the 96-well plate and the detection wavelength was 562 nm.

A solution with a concentration of 5% (*w*/*v*) of *LHPs* was prepared, and 1 mL was taken for HPLC analysis. Twenty-μL aliquots were injected onto an HPX-87 H column (300 × 7.8 mm, 5 μm, Bio-Rad, Hercules, CA, USA) with 5 mM H_2_SO_4_ as the mobile phase, at a detection wavelength of 210 nm [22].

### 2.6. Effects of LHPs on the Growth of Probiotics

To explore the effects of postbiotics obtained through spray drying on probiotics. The blank control group consisted of MRS medium without the addition of *LHPs*. After sterilization, the *LHPs* were added to MRS medium at final concentrations of 0.5, 1.0, 1.5, 2.0, and 2.5% (*w*/*v*). The activated probiotics were inoculated into the medium at an inoculum of 2% (*v*/*v*) and incubated at 37 °C. Absorbance values were measured at 2 h intervals during the incubation period using the growth curve protocol. The results correspond to three independent tests.

### 2.7. Assessment of the Antimicrobial Activity of LHPs

The Oxford cup method was used for the bacterial inhibition test [23]. A volume of 15–20 mL of agar medium was poured into the lower layer of the plate and allowed to cool and solidify. Next, the plate was placed in the Oxford cup. Then, the activated pathogenic bacteria (1 × 10^6^ CFU/mL) were inoculated into LB semi-solid medium. After it solidified, the Oxford cup was removed under sterile conditions. The medium was placed in the incubator and incubated at 37 °C for 18 h. After cultivation, the results were measured using a vernier caliper. The inhibitory diameter was expressed in millimeters [24].

Determination of Minimum Inhibitory Concentration of *LHPs*. The experiment was divided into four groups: A1, A2, A3, and A4. The activated pathogenic bacteria (10^6^ CFU/mL) were inoculated in LB medium, and then *LHPs* were added to it to give a concentration of 1%, 3%, 5%, 10%, and 20%. After culturing each group in a 37 °C incubator for 24 h, 200 μL of cell-free supernatant were collected and added to a 96-well plate. The OD_600_ value was measured using the microplate reader, with each sample being repeated three times. The formula for calculating the minimum inhibitory concentration of *LHPs* against two pathogenic bacteria is as follows:

(1) $$\mathrm{Inhibition}\;\mathrm{rate}\;(\%)=(1-\frac{A2-A1}{A4-A3})\times 100\%$$

Notes: A1: LB medium + different concentrations of *LHP* solutions; A2: pathogenic bacteria+ LB medium + different concentrations of *LHP* solutions; A3: LB medium; A4: pathogenic bacteria + LB medium.

The effect of protease on the antibacterial ability of *LHPs*. Papain (pH = 6.5), trypsin (pH = 7.5), and pepsin (pH = 2.0) were added separately to the prepared solutions to achieve a final enzyme concentration of 1mg/mL and determine the optimal pH for each enzyme. After enzymatic hydrolysis in a constant temperature water bath at 37 °C for 2 h, the enzymatic solution was adjusted back to its original pH value for in vitro antibacterial testing.

The effect of *LHPs* on the antibacterial ability under different pH levels. The solution pH was adjusted to 2.0, 3.0, 4.0, 5.0, 6.0, and 7.0, and the inhibition experiments were conducted and then the inhibition circle diameter was measured using a vernier caliper.

### 2.8. Digestion of LHPs in the SHIME Model

**Figure 1:**
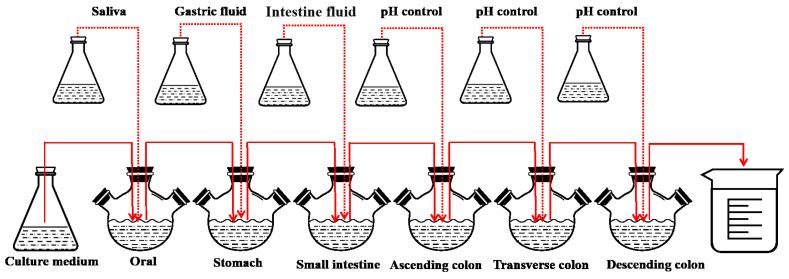
Schematic diagram of Simulated Human Intestinal Microbial Ecosystem (SHIME).

This experiment refers to the in vitro simulation of the human microbial digestive system SHIME model with slight improvements [25]. The SHIME model consists of six reactors: the oral, the stomach, the small intestine, and the ascending colon, transverse colon, and descending colon (Figure 1).

Before the experiment, fecal suspension was added to the last three reactors separately. Fecal suspensions from three healthy adult volunteers were respectively inoculated into the ascending colon (AC), transverse colon (TC), and descending colon (DC). The experimental subjects must have no history of gastrointestinal diseases, have not used antibiotics in the past six months, and have signed an informed consent form prior to participation in the study. Fresh fecal samples were collected and stored in anaerobic bags at 4 °C within a collection box. Fifty grams of feces were weighed and added to 250 mL of inoculation fluid (sterile PBS buffer, 0.1 M, pH = 7, with 1 g/L of sodium thioglycolate serving as a reductant). The mixture was homogenized and shaken evenly using a homogenizer and shaker. Then, it was centrifuged at 4 °C at 1000× *g* for 10 min. The supernatant was carefully collected and divided into 50 mL portions for AC, 50 mL for TC, and 60 mL for DC.

In this experiment, 20 min of nitrogen gas was introduced every 4 h to simulate the anaerobic environment of the human gastrointestinal tract, and the pH was adjusted by a pH controller (Uniontest Corp, Guangzhou, China). The pH levels (7.0~7.2, 2.0~2.5, 5.0~5.5, 5.6~5.9, 6.0~6.5, 6.6~6.9 in the oral, stomach, small intestine, ascending colon, transverse colon, and descending colon, respectively) of the six reactors were tuned to the pH environment of various parts of the human body. The experiment was divided into four phases: the basal period (2 weeks), the pathogenic period (1 day), the treatment period (2 weeks), and the washout period (2 weeks). Antioxidant measurements were taken on the last day of each phase. During the test period, 250 mL of sterile medium was added twice a day [26].

To simulate the digestive process, 50 mL of medium saliva, 100 mL of gastric fluid, and 60 mL of intestinal fluid were added to the oral, stomach, and small intestine cavities, respectively. Digestive fluids (Table 1) were composed of the corresponding enzymes and electrolytes. During the pathogenic phase, *E. coli* IQCC10126 and *S. aureus* ATCC13565 (10^6^ CFU/mL) were injected into each of the three reactors. After administering the bacteria, the peristaltic pumps were stopped, allowing the pathogenic bacteria to naturally colonize for 24 h before sampling. Throughout this period, an additional 2.5 g/d of postbiotics were incorporated into the culture medium.

### 2.9. Antioxidant Measurement

Determination of the changes in total antioxidant capacity during in the SHIME model using the DPPH method [27]. Samples were taken from each of the six reactors of the intestinal model (*LHPs* not added were replaced with skimmed milk powder of equivalent quality). The individual samples were diluted 10-fold with distilled water and mixed well; 100 μL of the samples and 100 μL (0.1 mmoL/L) of DPPH solution in ethanol were added to a 96-well plate, and the reaction was carried out at 37 °C for 30 min protected from light, and absorbance values were measured at 517 nm. Blank group A0 was replaced with deionized water and control group A1 was replaced with anhydrous ethanol for DPPH ethanol solution. The formula for calculating DPPH radical scavenging rate is as follows:

(2) $$\mathrm{Inhibition}\;\mathrm{rate}\;(\%)=(1-\frac{A1-A2}{A0})\times 100\%$$

Notes: A0: absorbance of 100 μL DPPH ethanol solution + 100 μL water; A1: absorbance of 100 μL DPPH ethanol solution + 100 μL sample; A2: absorbance of 100 μL anhydrous ethanol solution + 100 μL sample.

### 2.10. Intestinal Microbiota Analysis

Samples were taken from each colon and placed in cryovials, immediately placed in liquid nitrogen, and then transferred to a −80 °C freezer for storage. Dry ice was used to transport the samples to Botai Biotechnology Co., Ltd. (Shanghai, China) for 16S rDNA gene amplicon sequencing. The bioinformatics analysis of sequencing data was conducted using the Quantitative Insights Into Microbial Ecology (QIIME 2) software.

### 2.11. Determination of SCFAs

The 5 mL sample was taken in the model and centrifuged (13,000× *g*, 5 min). After centrifugation, the samples were filtered through a 0.45 μm filter membrane and the supernatant was then analyzed by high-performance liquid chromatography. Five mM H_2_SO_4_ was employed as the mobile phase. A 20 μL aliquot was injected into an HPX-87 H column (300 × 7.8 mm, 5 μm, Bio-Rad Corp, CA, USA) where the detected wavelength of the column was 210 nm [28].

### 2.12. Statistical Analysis

All data were obtained from three parallel experiments, and the data were expressed as mean ± standard deviation (SD). The graphing tool was Origin 2021. The analysis tool was IBM SPSS Statistics 20. *p* < 0.05 indicates statistical significance.

## 3. Results

### 3.1. Survivability Testing of LHPs

After passaging and cultivation, the viable cell count of *L. helveticus* reached 1 × 10^9^ CFU/mL. However, when the *LHPs* were inoculated onto MRS solid medium, no colonies were observed on the agar plates. This indicates that the *L. helveticus* had been completely inactivated.

### 3.2. Composition Analysis of LHPs

The content of the major components in *LHPs* is polysaccharide, protein, and SCFAs. The content of polysaccharide was 235.69 ± 0.26 mg/g, while the content of protein was 212.00 ± 0.02 mg/g. Short chain fatty acids included acetic acid (13.9 mg/g), propionic acid (6.5 mg/g), butyric acid (0.2 mg/g), isobutyric acid (0.2 mg/g), and valeric acid (4.3 mg/g).

### 3.3. Effect of LHPs on the Growth of Probiotic Bacteria

**Figure 2:**
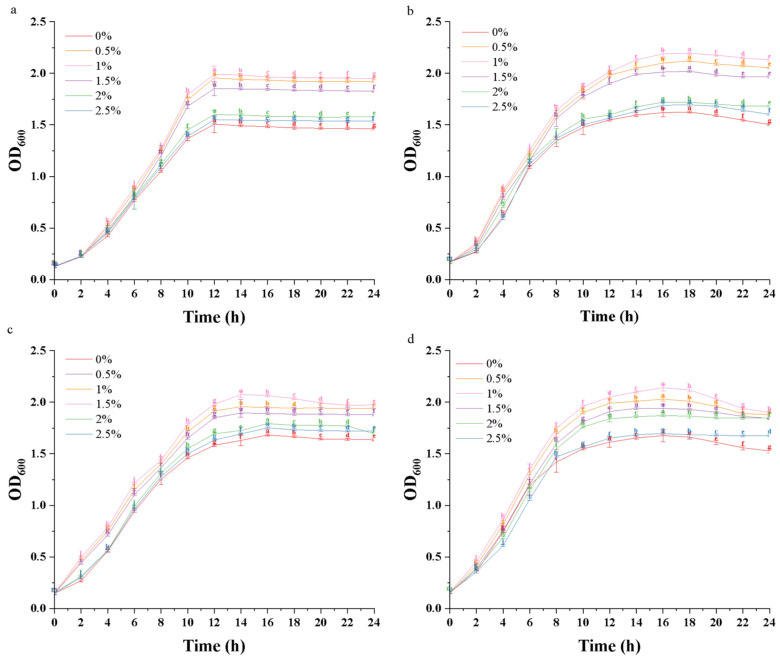
Effect of *LHPs* on probiotic growth curves. ((**a**) *L. plantarum* DNZ-4, (**b**) *L. lactis* L1, (**c**) *L. casei* 0.8072, (**d**) *L. rhamnosus* L08). Note: The same letter indicates no significant difference between groups (*p* < 0.05), while different letters indicate a significant difference between groups (*p* > 0.05).

The effects of *LHPs* on the growth curves of the four probiotic species are illustrated in Figure 2. Varying concentrations of *LHPs* had significant effects on the growth curves of the four probiotic species. From 0 to 4 h, the growth of *L. plantarum* DNZ-4 was not significantly affected by the presence of *LHPs* (*p* > 0.05). However, during the logarithmic and stable phases, the addition of different concentrations of *LHPs* had a significant impact on its growth (*p* < 0.05). Specifically, at a concentration of 1% of *LHPs*, the viable microbial count of *L. plantarum* reached its peak. The other three probiotics (*L. lactis* L1, *L. casei* 0.8072, *L. rhamnosus* L08) exhibited a similar trend. During the lag phase, the growth of these probiotics was not significantly affected by the presence of *LHPs* (*p* > 0.05). However, after entering the stable phase following logarithmic growth, significant differences in OD_600_ values were observed (*p* < 0.05). The optimal concentrations for promoting growth in each group were 1%, 1%, 1.5%, and 1%, respectively.

### 3.4. Bacteriostatic Activity of LHPs

**Figure 3:**
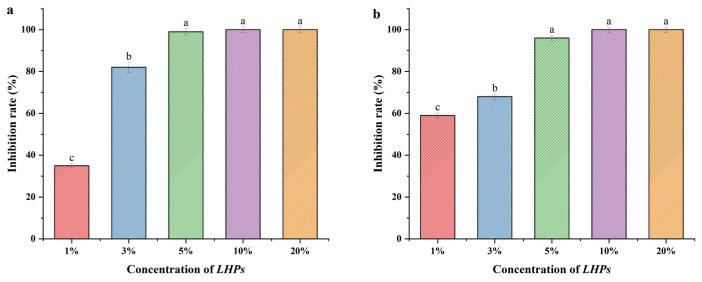
MIC of *LHPs*. ((**a**) Inhibition rate of *LHPs* against *E. coli* IQCC10126; (**b**) Inhibition rate of *LHPs* against *S. aureus* ATCC13565). Note: The same letter indicates no significant difference between groups (*p* < 0.05), while different letters indicate a significant difference between groups (*p* > 0.05). (The same as above).

In this experiment, the Oxford cup method was used for the evaluation of bacterial inhibitory ability, as shown in Figure 3. The *LHPs* at concentrations of 1% and 3% showed significant differences compared to 5% (*p* > 0.05), while there was no significant difference among 5%, 10%, and 20% concentrations of *LHPs* (*p* < 0.05). In summary, the results indicated that a 5% concentration of *LHPs* achieved a 99% inhibition rate against both *S. aureus* ATCC13565 and *E. coli* IQCC10126. This demonstrates that the MIC of *LHPs* is 5%.

The bacteriostatic activity of *LHPs* produced by different processing methods is shown in Table 2. The results show that the antibacterial activity of *LHPs* produced by different processing methods had a significant effect on both *S. aureus* ATCC13565 and *E. coli* IQCC10126 (*p* < 0.05). The spray-dried postbiotics reached the maximum size of the circle of inhibition against *S. aureus* ATCC13565 and *E. coli* IQCC10126. In the spray-drying treatment, the inhibition of trypsin-treated postbiotics on *E. coli* IQCC10126 was significantly reduced (*p* < 0.05), and there was no significant effect on *S. aureus* ATCC13565. After treatment with pepsin, the inhibitory ability of postbiotics against *E. coli* IQCC10126 and *S. aureus* ATCC13565 was significantly reduced (*p* < 0.05). The inhibitory ability of postbiotics treated with papain and catalase (H_2_O_2_) was significantly reduced (*p* < 0.05) against *E. coli* IQCC10126 but had no significant effect on the inhibitory ability against *S. aureus* ATCC13565.

The impact of different pH conditions on the inhibitory abilities of *LHPs* is shown in Table 3. It can be seen that the circle of inhibition of both pathogenic bacteria by the postbiotics treated by the four different treatments shrinks sharply with increasing pH, and the inhibitory ability is almost zero when the pH is 7. The pH stability analysis showed that *LHPs* had better antimicrobial activity under acidic conditions. It can be seen that the antimicrobial substances in *LHPs* are both proteinaceous and organic acids.

### 3.5. Changes in Total Antioxidant Capacity in the SHIME Model

**Figure 4:**
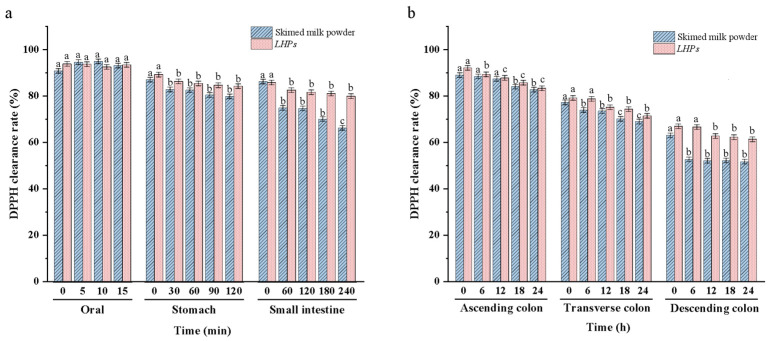
Changes in DPPH clearance rates, (**a**) in the mouth, stomach, small intestine; (**b**) in the colon. Note: The same letter indicates no significant difference between groups (*p* < 0.05), while different letters indicate a significant difference between groups (*p* > 0.05).

The addition of *LHPs* affected antioxidant capacity in the SHIME model across six reactors (Figure 4). In the oral reactor, DPPH clearance rates were higher with postbiotics compared to skimmed milk powder, though not significantly (*p* > 0.05). In the stomach reactor, the clearance rate decreased significantly from 89.20% to 86.31% within 30 min (*p* < 0.05) but remained stable from 30 to 120 min (*p* > 0.05). In the small intestine reactor, the rate dropped from 85.83% to 82.50% between 0–60 min, indicating a change in antioxidant capacity (*p* < 0.05), but no significant changes were seen from 60 to 240 min (*p* > 0.05). In the colon reactors, the presence of gut microbiota improved antioxidant activity. At 0 h, DPPH clearance rates were highest in all three colon regions. The ascending colon’s antioxidant capacity significantly decreased over 0–18 h (*p* < 0.05) but stabilized after that (*p* > 0.05). The transverse and descending colons showed significant decreases within the first 6 h (*p* < 0.05) but remained stable afterward (*p* > 0.05). Compared to the control group with skimmed milk powder, the clearance rates at 24 h increased in the ascending (from 82.70% to 83.31%), transverse (from 68.90% to 71.43%), and descending colons (from 51.66% to 61.37%). This indicates that postbiotics resulted in a higher overall antioxidant capacity in the colon than the control.

### 3.6. Changes in Gut Microbiota Composition

#### 3.6.1. Analysis of the Diversity of Intestinal Flora

**Figure 5:**
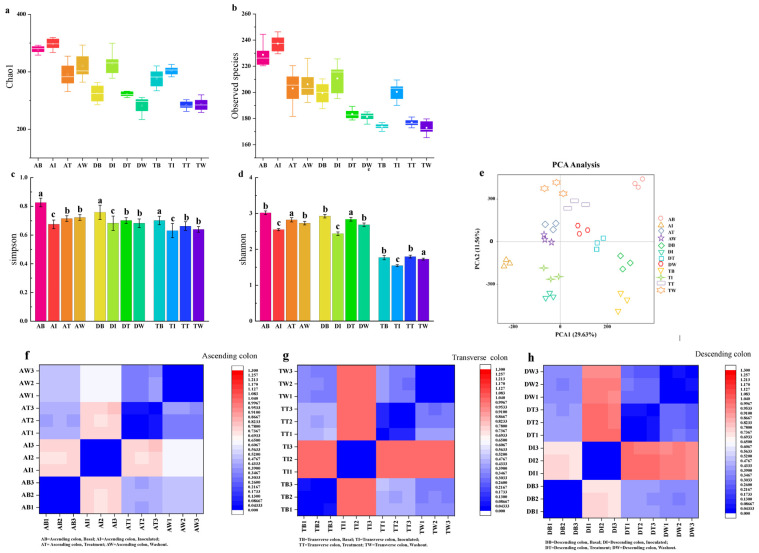
Diversity analysis of intestinal flora. (**a**) Chao 1 index, (**b**) Observed Species index, (**c**) Shannon index, (**d**) Simpson index, (**e**) PCA analysis, (**f**) ascending colon, (**g**) transverse colon, (**h**) descending colon. Note: The same letter indicates no significant difference between groups (*p* < 0.05), while different letters indicate a significant difference between groups (*p* > 0.05).

Alpha diversity assesses species diversity in single samples, using indices such as Chao1, Observed Species, Shannon, and Simpson to evaluate microbial community diversity in the colon. Figure 5a–d presents the alpha diversity analysis of gut microbiota in three colon samples. During the basal period, the Chao1 indices were 338.94, 262.12, and 288.72. After adding pathogenic bacteria, Chao1 significantly increased (*p* < 0.05) to 348.23, 314.69, and 301.97. Treatment with *LHPs* then significantly decreased Chao1 (*p* < 0.05) to 295.08, 261.01, and 241.74. The observed species index showed a similar trend, increasing after pathogenic bacteria addition (*p* < 0.05) from 228.82, 199.36, and 173.84 to 237.36, 183.42, and 200.46, and then decreasing during treatment with values of 203.02, 183.42, and 176.86 (*p* < 0.05). The Shannon and Simpson indices reflect species diversity, with higher values indicating greater diversity. During the basal period, Shannon indices were 3.01, 2.92, and 1.77. These significantly decreased (*p* < 0.05) after pathogenic bacteria addition to 2.55, 2.43, and 1.55. Shannon values then significantly increased (*p* < 0.05) during postbiotics treatment to 2.82, 2.83, and 1.79. Simpson indices during the basal period were 0.83 ± 0.027, 0.76 ± 0.029, and 0.70 ± 0.011. After pathogenic bacteria addition, Simpson values significantly decreased (*p* < 0.05) to 0.67 ± 0.020, 0.68 ± 0.011 and 0.63 ± 0.027, but increased significantly (*p* < 0.05) after postbiotics treatment to 0.71 ± 0.023, 0.70 ± 0.027 and 0.66 ± 0.012. No significant changes occurred during the washout period.

As illustrated in Figure 5e, the principal component analysis (PCA) of the intestine demonstrates that similar samples cluster together while showing distinct separation at different stages. The contribution rates of the first two principal components are 29.63% and 11.56%, respectively. Following the inoculation with pathogenic bacteria, significant changes occurred in the gut microbiota, with all three reactors of the colon shifting to the left and clustering in the second and third quadrants. Notably, there are significant differences observed between the treatment period and the pre-treatment phase, with the concentration of gut microbiota in the three colon reactors moving towards the right. The washout period exhibited a pattern similar to that of the treatment period.

Beta diversity analysis compares species diversity across samples from four periods. Figure 5f–h displays the weighted normalized diversity distance heatmap of the three sections of the colon at these stages. In the ascending, transverse, and descending colons, the UniFrac values during the *LHP* treatment and pathogenic bacteria periods ranged from 0.73–0.75, 0.98–0.99 and 0.96–0.98, respectively, indicating significant species differences (red color). This suggests a high similarity in microbial community structure between these two periods. In contrast, during the treatment with postbiotics and washout periods, UniFrac values ranged from 0.31 to 0.33 in the ascending colon, 0.27 to 0.30 in the transverse colon, and 0.33 to 0.39 in the descending colon, indicating minimal species differences and high uniformity (blue color) between these periods.

#### 3.6.2. Changes in the Abundance of Intestinal Flora

**Figure 6:**
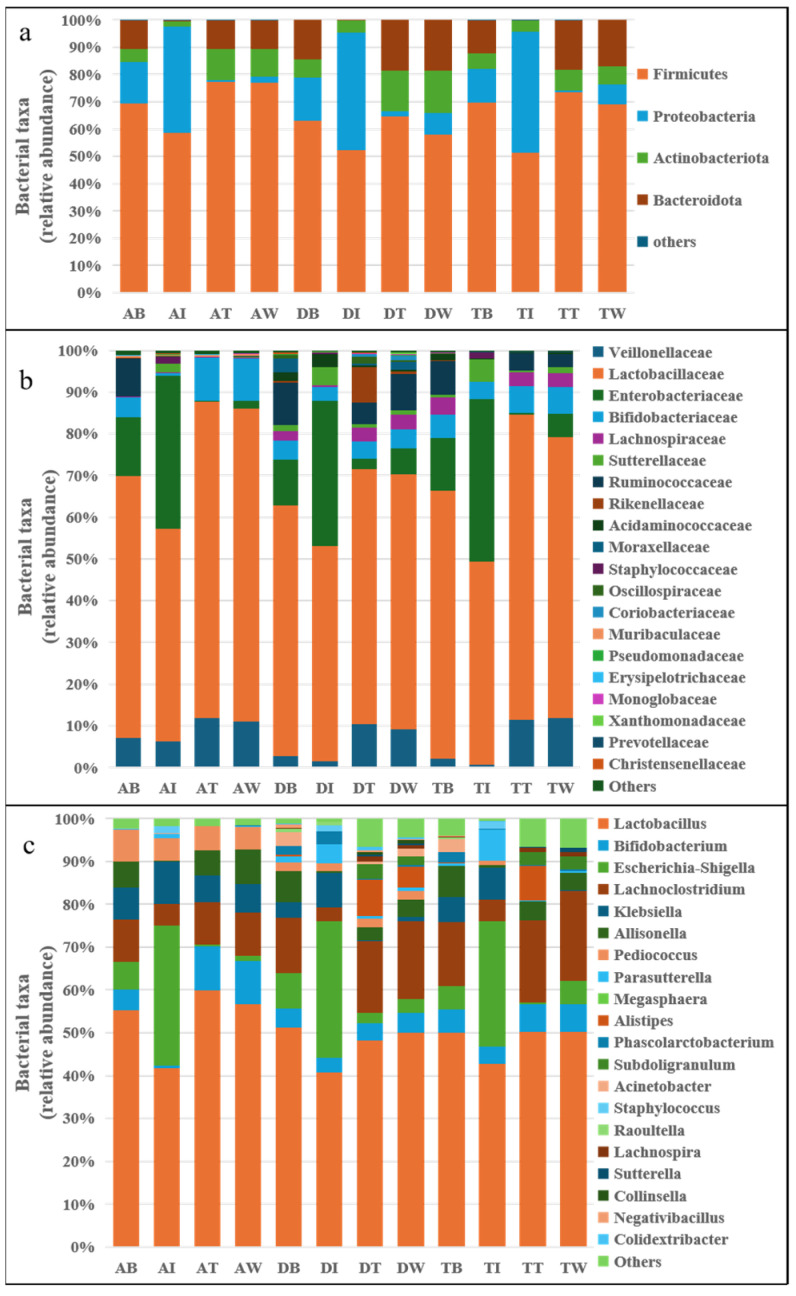
Changes in gut microbiota during in vitro simulated digestion. (**a**) At the phylum level; (**b**) at the family level; (**c**) at the genus level). Note: AB, AI, AT, AW, TB, TI, TT, TW, DB, DI, DT, and DW’s annotations are the same as above.

Figure 6a shows the changes in gut microbiota at the phylum level in three colon reactors over four periods. During the stable period, *Firmicutes* was the dominant phylum in all three reactors. After the addition of *E.coli* IQCC10126 and *S. aureus* ATCC13565, the relative abundance of *Firmicutes* decreased while that of *Proteobacteria* increased significantly. Specifically, *Proteobacteria* rose from 14.96%, 15.90%, and 12.46% to 39.02%, 43.22%, and 44.45%, respectively. During the treatment period, the abundance of *Proteobacteria* significantly decreased (*p* < 0.05), while the abundance of *Firmicutes* significantly increased, rising from 58.53%, 52.18%, and 51.31% to 77.17%, 64.62%, and 73.30%. There were no significant changes observed during the washout period.

Figure 6b shows the changes in gut microbiota at the family level over four periods. During the stable period, *Lactobacillaceae* and *Bifidobacteriaceae* were the dominant bacterial families in the three colon reactors. After the introduction of pathogenic bacteria, the relative abundance of *Lactobacillaceae* and *Bifidobacteriaceae* significantly decreased, while that of *Enterobacteriaceae* and *Sutterellaceae* significantly increased (*p* < 0.05). Specifically, *Lactobacillaceae* abundance decreased from 62.77%, 60.11%, and 69.14% to 50.94%, 51.62%, and 48.65%, while *Enterobacteriaceae* increased from 14.19%, 10.96%, and 12.66% to 38.57%, 34.67%, and 34.03%. After administering postbiotics for treatment, the relative abundance of *Enterobacteriaceae* significantly decreased, while that of *Lactobacillaceae*, *Bifidobacteriaceae, and Lachnospiraceae* increased significantly.

To investigate key differences in gut flora structure, the 20 genera with the highest relative abundance were compared. The abundance of intestinal flora varied during the four periods across the three colon reactors (Figure 6c). After inoculation with pathogenic bacteria, significant changes were observed; the relative abundance of *Escherichia*, *Klebsiella*, *Parasutterella* and *Staphylococcus* increased significantly (*p* < 0.05), while that of *Lactobacillus*, *Bifidobacterium*, *Allisonella,* and *Pediococcus* decreased significantly (*p* < 0.05). During the treatment period with *LHPs*, the abundance of beneficial genera such as *Lactobacillus*, *Bifidobacterium*, and *Pediococcus* increased significantly (*p* < 0.05), while the abundance of pathogenic genera like *Escherichia*, *Parasutterella*, and *Staphylococcus* decreased significantly (*p* < 0.05). There were no significant fluctuations in the abundance of any bacterial genus during the washout period.

### 3.7. Effect of LHPs on SCFAs Production

To investigate the impact of *LHPs* on microbial activity, SCFAs were measured in three colon tissues. The study illustrates changes in SCFA content throughout the simulated digestion process, with acetic acid, propionic acid, and isobutyric acid identified as the main metabolites. During the basal period, six types of SCFAs were produced in all three colon tissues. After introducing pathogenic bacteria, total fatty acid content in the three colon tissues significantly decreased (*p* < 0.05): from 99.40 mmol/L, 125.88 mmol/L, and 135.71 mmol/L to 84.27 mmol/L, 103.55 mmol/L, and 121.75 mmol/L, respectively. Following treatment with postbiotics, total fatty acid content significantly increased (*p* < 0.05) to 168.58 mmol/L, 175.42 mmol/L, and 205.50 mmol/L in the ascending, transverse, and descending colons, respectively. Although there was a downward trend during the washout period, total fatty acid levels remained significantly higher compared to the basal period (*p* < 0.05), reaching 113.47 mmol/L, 136.15 mmol/L, and 156.97 mmol/L, respectively. The introduction of pathogenic bacteria reduced intestinal flora diversity, leading to decreased SCFAs production. However, during the treatment period, *LHPs* were utilized by the gut microbiota to enhance the production of SCFAs.

## 4. Discussion

Postbiotics are inanimate microorganisms and/or their components that are beneficial to the host’s health. They include a variety of components, such as inactivated microbial cells, cell wall components, functional proteins, peptides, SCFAs, polyamines, vitamins, bacteriocins, and other bioactive metabolites. This experiment measured the total sugar and total protein contents in *LHPs* and performed qualitative and quantitative analysis of short-chain fatty acids. *L. helveticus*, as a member of the *Lactobacillus* within the *Firmicutes*, is known for its effective production of organic acids such as acetic acid, propionic acid, butyric acid, isobutyric acid, and valeric acid through metabolic processes [29]. Due to its gram-positive nature and thick cell wall, *L. helveticus* has a higher concentration of peptidoglycans. *LHPs* were produced by spray drying, which dehydrated *L. helveticus* and allowed extracellular polysaccharide to flow out [30]. During fermentation of skimmed milk powder, *L. helveticus* breaks down lactose to produce lactose and galactose and may also contain small amounts of surface-attached polysaccharides like glucose and arabinose, along with proteins that are challenging to remove. Further research is warranted, given that most postbiotics are peptides or peptide precursors, which underscores the potential of *L. helveticus* to inhibit pathogenic bacteria.

Emerging research highlights the strong antibacterial properties of postbiotics. For instance, *L. plantarum* M4L1, known for producing bacteriocins, exhibits exceptional antibacterial capabilities. This study focused on the antibacterial effects of *LHPs* against *E.coli* IQCC10126 and *S. aureus* ATCC13565 under various conditions, finding the MIC to be 5%. Among different inactivation methods, spray drying was the most effective in preserving the antibacterial substances without compromising their functionality. Additionally, our study revealed that the antimicrobial substances in *LHPs* are sensitive to pepsin and exhibit limited antibacterial activity under near-neutral conditions; however, their effectiveness is enhanced in acidic environments. The cell-free supernatant (CFS) of *L. acidophilus* LA5 contained protease-sensitive antibacterial substances, including bacteriocins and antimicrobial enzymes [31]. Postbiotics are effective antimicrobial agents, with their bactericidal and bacteriostatic effects attributed to the synergistic action of multiple molecules such as organic acids, peptides, SCFAs [32]. Postbiotics produced from *L. plantarum* and inulin have been shown to inhibit various pathogens, and *L. plantarum* M.2 can prevent the formation of pathogenic biofilms [33,34].

Moreover, *LHPs* can support the growth of other probiotics, with optimal concentrations for *L. plantarum*, *L. lactis*, *L. casei*, and *L. rhamnosus* at 1%, 1%, 1.5%, and 1%, respectively. This effect is likely due to the rich content of polysaccharides and lactic acid in postbiotics, which promote probiotic growth. Consistent with our findings, *Pichia pastoris* postbiotics have also been shown to enhance the growth of *Lactobacillus* FP13, aligning with the findings of this study [35].

Antioxidant capacity is a crucial biological activity of *LHPs*. Previous studies have identified several cellular metabolites with antioxidant properties in these postbiotics, including bioactive peptides and polysaccharides [36,37]. Using the DPPH assay, our experiments demonstrated that the antioxidant capacity remains relatively stable in the oral reactors but declines as the postbiotics move into the stomach and small intestine reactors. A marked drop in antioxidant capacity in the colon suggests that the polysaccharides in postbiotics are effectively absorbed and utilized by the gut microbiota, correlating with this decrease. Notably, the antioxidant rate of the postbiotics group is significantly higher than that of the control group, aligning with findings reported by Humam [38].

The human body provides a stable, nutrient-rich environment for microorganisms, which promotes microbial activity and regulates intestinal homeostasis [39]. The study indicates that postbiotics play a unique role in regulating gut microbiota composition. At the phylum level, *Bacteroidetes* and *Firmicutes* account for over 90% of the gut microbiota. During pathogenic infections, the abundance of *Salmonella* and *E. coli* increased. However, this abundance decreased with the addition of postbiotics, suggesting they can inhibit pathogenic growth. At the family level, in the SHIME model infected with pathogenic bacteria, the relative abundance of *Enterobacteriaceae* and *Sutterellaceae* significantly decreased after adding postbiotics, while *Bifidobacteriaceae* and *Lachnospiraceae* increased. *LHPs* can enhance microbial communities that produce SCFAs, such as butyrate-producing *Bifidobacteriaceae* and *Lachnospiraceae* [40,41]. At the genus level, postbiotics increased beneficial bacteria while reducing harmful ones, indicating effective inhibition of *E.coli* and *S. aureus*. The high lactic acid content in *LHPs* aided in suppressing harmful bacteria, while the relative abundance of beneficial *Lactobacillus* and *Bifidobacterium* increased. Antibacterial compounds in postbiotics help control the growth of pathogens, maintaining gut ecosystem balance [42]. Postbiotics contain SCFAs, proteins, peptides, hormones, and neuroactive compounds, which selectively promote probiotic growth and enhance beneficial bacterial genera [43]. *Bifidobacterium*, an important probiotic in the gut, helps regulate defense responses and prevent infections [44]. *LHPs* stimulate *Bifidobacterium* growth, aligning with findings from Warda’s research [45]. Gene sequencing of 16S rRNA was conducted to assess the impact of *LHPs* on the alpha and beta diversity of gut microbiota. After introducing pathogenic bacteria into the SHIME model, the Chao1 index significantly increased, indicating that pathogenic bacteria disrupt the balance of gut microbiota and lead to a higher total number of species. However, the Shannon and Simpson indices significantly decreased, showing reduced gut microbiota diversity caused by the presence of pathogenic bacteria. With the addition of postbiotics, both the Chao1 index and observed species index significantly decreased, while the Shannon and Simpson indices increased, highlighting the positive effect of postbiotics in enhancing gut microbiota diversity and richness. During the washout period, all four indices remained stable, confirming the lasting impact of postbiotics on maintaining intestinal microbiota stability. In terms of beta diversity, UniFrac values, which indicate species differences, were highest during the pathogenic and therapeutic phases, suggesting significant species differences. In contrast, the UniFrac values during the treatment and stable phases indicate that the addition of postbiotics can effectively balance the gut microbiota. The values during the treatment and washout periods suggest that postbiotics exert a sustained therapeutic effect on gut microbiota. Other studies have shown that endogenous preparations can enhance the richness and diversity of infants’ gut microbiota, supporting the findings of this study [46]. Overall, these results suggest that postbiotics have the potential to regulate intestinal health and enhance microbial diversity. SCFAs are crucial for maintaining intestinal homeostasis and reflect changes in microbial communities.

SCFA levels significantly decreased after introducing pathogenic bacteria, likely due to disruptions in gut microbiota diversity and ecosystem balance [47]. Once the treatment period began, SCFA levels significantly increased, suggesting that beneficial bacteria, alongside postbiotics rich in SCFAs, enhance SCFA production in the intestine. Probiotics help restore and maintain a balanced gut microbiota by producing SCFAs, which improve digestion, nutrient absorption, and overall gut health [48]. Inactive *Lactobacilli* can also influence SCFA levels and gut microbiota composition [49]. Moreover, *LHPs* can promote the growth of probiotics, such as *Bifidobacteria*, by serving as fermentable substrates, leading to changes in metabolic profiles and interactions among intestinal bacteria [50].

## 5. Conclusions

This experiment employed the SHIME in vitro digestion model to investigate the total antioxidant capacity of *LHPs*, and to thoroughly analyze their impact on intestinal infections caused by pathogenic bacteria. Furthermore, the study delved into the primary constituents of postbiotics and explored the regulatory effects of *LHPs* on both probiotics and pathogenic bacteria. In summary, *LHPs* demonstrated a positive promotional effect on the growth of probiotics. Moreover, *LHPs* produced through spray-drying technology exhibited high antibacterial properties, with their efficacy primarily due to the synergistic action of proteins and acids. *LHPs* also significantly enhanced the total antioxidant capacity in the colon and effectively suppressed the proliferation of intestinal pathogenic bacteria, a response closely linked to their rich content of organic acids. Additionally, postbiotics markedly increased the relative abundance of beneficial bacterial groups, such as *Lactobacillus* and *Bifidobacterium*, while decreasing the relative abundance of potentially pathogenic bacteria, including *Escherichia*, *Klebsiella*, and *Parasutterella*. Furthermore, the introduction of postbiotics fostered the production of SCFAs in the gut, which positively influenced the regulation of gut microbiota balance. The SHIME model, as an important in vitro research tool, plays a significant role in gut microbiome studies. However, to gain a more comprehensive understanding of the complexity of the intestinal microbiota and provide more reliable evidence for disease prevention and treatment, it is necessary to combine various approaches such as animal models, clinical studies, and multi-omics analyses, while continuously improving and refining the existing research methods.

## Figures and Tables

**Table 1:** Composition of various digestive fluid components.

Composition	Content
Saliva	
α-amylase (1500 U/mg)	7.5 mg
Mucin	100.0 mg
Salivary electrolyte	50 mL
Gastric fluid	
Pepsin (3000 U/mg)	23.6 mg
Lipase	25.0 mg
CH_3_COONa	1 mL
Gastric electrolyte	100 mL
Intestinal fluid	
Trypsin (3000 U/mg)	6.5 mg
Bile salt	200 g (4%, *w*/*w*)
Pancreatic enzyme solution	50 g (7%, *w*/*w*)
Intestinal electrolytes	50 mL

**Table 2:** Effect of different treatments on the bacteriostatic capacity of *LHPs*.

Treatment	Inhibitory Diameter (mm)					
	Strains	Sample Solution	Trypsin	Pepsin	Papain	Catalase
Spray drying	*E. coli* IQCC10126	13.02 ± 0.26	11.17 ± 0.26 *	10.32 ± 0.92 *	11.54 ± 0.36 *	10.16 ± 0.11 *
	*S. aureus* ATCC13565	13.85 ± 0.74	11.96 ± 0.83	11.29 ± 0.61 *	12.87 ± 0.28	13.02 ± 0.06
Freeze drying	*E. coli* IQCC10126	9.22 ± 0.07	8.22 ± 0.09 **	8.42 ± 0.27 *	8.24 ± 0.21 *	8.75 ± 0.14 *
	*S. aureus* ATCC13565	12.92 ± 0.47	11.80 ± 0.10	10.30 ± 0.14 *	10.07 ± 0.46 *	12.66 ± 0.51

Note: In the table, * indicates a significant difference, * = *p* < 0.05, ** = *p* < 0.01.

**Table 3:** Inhibition diameters obtained from inhibition experiments with different pH on *LHPs*.

Inhibitory Diameter (mm)							
	Strains	pH2.0	pH3.0	pH4.0	pH5.0	pH6.0	pH7.0
Spray drying	*E.coli* IQCC10126	19.80 ± 0.44 ^a^	17.74 ± 0.24 ^b^	11.68 ± 0.16 ^c^	11.0 ± 0.16 ^c^	10.52 ± 0.11 ^d^	8.94 ± 0.04 ^e^
	*S. aureus* ATCC13565	18.84 ± 0.26 ^a^	12.32 ± 0.18 ^b^	11.58 ± 0.13 ^c^	9.96 ± 0.09 ^d^	8.84 ± 0.02 ^e^	8.00 ± 0.00 ^e^
Freeze drying	*E.coli* IQCC10126	18.83 ± 0.34 ^a^	14.58 ± 0.21 ^b^	10.0 ± 0.03 ^c^	9.92 ± 0.02 ^c^	9.42 ± 0.02 ^c^	8.00 ± 0.01 ^d^
	*S. aureus* ATCC13565	16.12 ± 0.25 ^a^	10.98 ± 0.16 ^b^	9.78 ± 0.09 ^c^	8.98 ± 0.04 ^d^	8.56 ± 0.02 ^d^	8.00 ± 0.01 ^e^

Note: The same letter indicates no significant difference between groups (*p* < 0.05), while different letters indicate a significant difference between groups (*p* > 0.05).

## Data Availability

The original contributions presented in the study are included in the article, further inquiries can be directed to the corresponding authors.
